# Endovascular therapy for acute basilar artery occlusion caused by vertebral artery dissection: Case report

**DOI:** 10.1097/MD.0000000000027995

**Published:** 2021-11-24

**Authors:** Changchun Jiang, Jiahui Liu, Jinfeng Zhang, Yujuan Cui, Junfeng Yang, Fei Hao, Yu Fan, Jianqi Wei

**Affiliations:** a Department of Neurology, Baotou Central Hospital, Baotou, Inner Mongolia, China; b Neurointerventional medical center of Inner Mongolia Medical University, Inner Mongolia, China; c Graduate School of Inner Mongolia Medical University, Inner Mongolia, China.

**Keywords:** acute ischemic stroke, anticoagulation, basilar artery occlusion, endovascular treatment, vertebral artery dissection

## Abstract

**Rationale:**

The best endovascular therapy revascularization strategies for acute ischemic stroke caused by vertebral artery dissection (VAD) are unclear. We describes a case of basilar artery (BA) occlusion caused by extracranial VAD, in which we used a stent-retriever to achieve thrombectomy in the BA through the contralateral vertebral artery (VA).

**Patient concerns:**

A 32-year-old male presented with a sudden-onset headache accompanied by articulation disorder, left-sided weakness, and tinnitus in the left ear.

**Diagnosis:**

Digital subtraction angiography showed the V1 to V2 segment dissection of the left VA and occlusion of the BA.

**Interventions:**

Thrombectomy was performed through the thinner right VA with three passes of the Solitaire FR device 4 × 20 mm in the BA, and angiograms showed modified treatment in cerebral ischemia 3 reperfusion of BA and left VA V4 segment still occluded.

**Outcomes:**

The patient had a modified Rankin Scale of 2 at 90 days, and re-established blood flow of the left VA and BA.

**Lessons:**

When extracranial VAD complicated with BA occlusion, choosing the clean-road path to perform a BA thrombectomy may be a fast and effective treatment strategy.

## 1 Introduction

Cervical artery dissection is an important cause of stroke in young patients.^[1]^ Vertebral artery dissection (VAD) has an annual incidence of 1 to 1.5 per 100,000 people.^[2]^ VAD with acute tandem proximal vertebral artery (VA) occlusion and concomitant major embolic basilar artery (BA) occlusion can lead to the onset of ischemic stroke with devastating consequences. In recent years, endovascular therapy (EVT) has become an effective method for the treatment of acute large vessel occlusion.^[3]^ However, the benefits of EVT revascularization strategies for acute ischemic stroke (AIS) caused by VAD are unclear. We report a case of BA occlusion caused by extracranial VAD, in which we used a stent-retriever to achieve mechanical thrombectomy in the BA through the contralateral VA.

## 2 Case report

**Figure 1:**
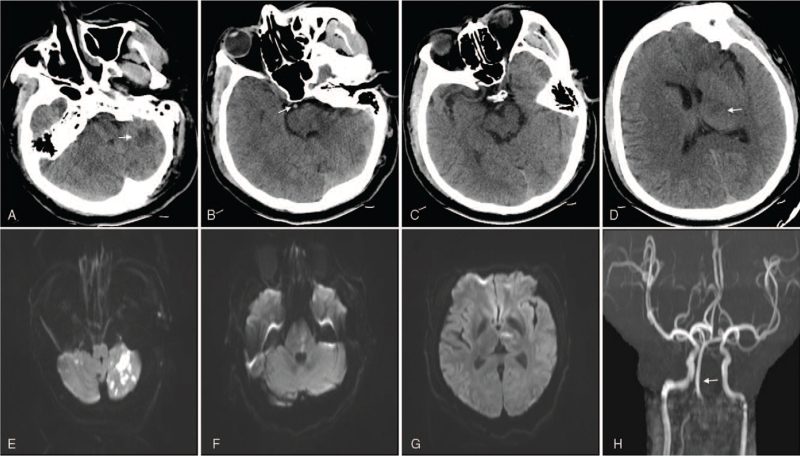
Baseline computed tomography revealed early signs of acute cerebral infarction of the left cerebellar hemisphere (A) and left thalamus (D) (white arrow). Hyperdense basilar artery sign was found (B), but no acute infarction was found in the midbrain (C). Magnetic resonance 24-hour after thrombectomy showed infarcted lesions of the bilateral cerebellum (E), pons (F) and left thalamus (G); blood flow recanalization of the basilar artery (H).

A 32-year-old male presented with a sudden-onset headache accompanied by articulation disorder, left-sided weakness, and tinnitus in the left ear. There was no history of trauma or common risk factors of cerebrovascular disease, including hypertension, diabetes, coronary heart disease, hypercholesterolemia, and smoking. In addition, there was no notable family history. Ultrasonography of cervical artery showed membrane echo floating in the V1 to V2 segments of the left VA, the diameter of the true lumen was 0.9 mm, the diameter of the false lumen was 3.7 mm, and no blood flow signal was detected at the distal end of the left V2 segment. His condition suddenly worsened after 20 hours, showing convulsions that lasted for approximately 7 minutes, followed by a decreased level of consciousness. The patient was transferred from a primary stroke center to our hospital after 5.5 hours of symptom aggravation. On admission, the patient had clonic limb convulsions, impaired consciousness of the Glasgow Coma Scale score of 9, and a National Institute of Health Stroke Scale score of 19 was recorded. Noncontrast computed tomography revealed early signs of acute cerebral infarction in the left cerebellar hemisphere and the left thalamus; a hyperdense BA sign was found also found (Fig. 1). The time of onset was more than 4.5 hours, so no intravenous thrombolysis was performed.

**Figure 2:**
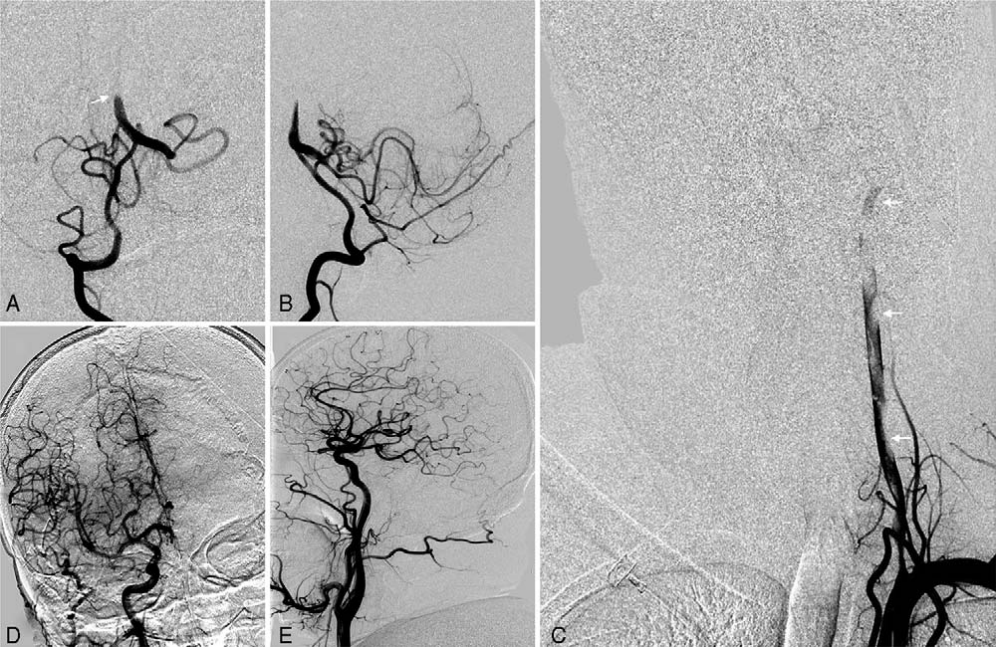
Cerebral digital subtraction angiography: occlusion of the middle portion of the basilar artery (A) (B); V1 to V2 segment dissection of the left vertebral artery (C); the right posterior communicating artery supplies blood to the right posterior cerebral artery.

Cerebral digital subtraction angiography (DSA) with conscious sedation revealed the V1 to V2 segment dissection of the left VA. There was no contrast agent filling at the distal of the V2 segment. DSA via the right VA revealed occlusion of the middle portion of the BA and V4 segment of the left VA. The American Society of Interventional and Therapeutic Neuroradiology and Society of Interventional Radiology grade was 0 (Fig. 2).

**Figure 3:**
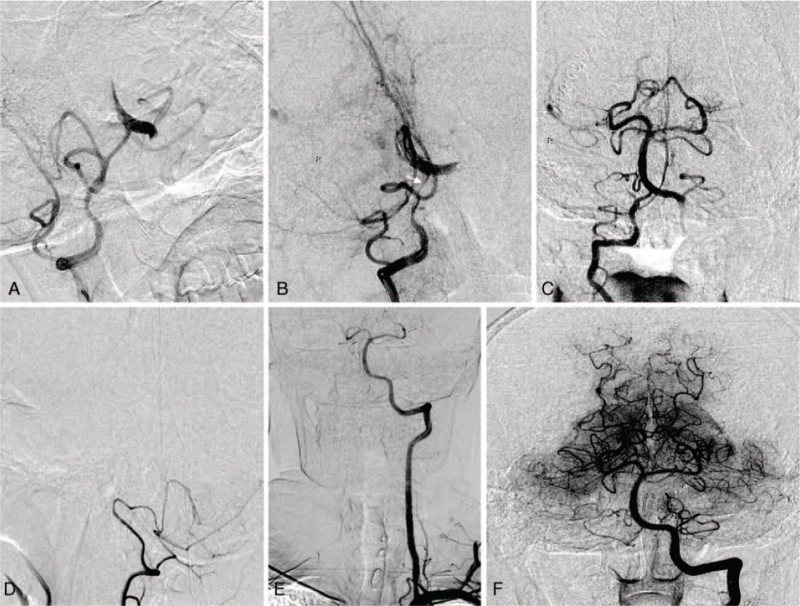
Endovascular treatment: (A) the first pass of stent retriever; (B) vasospasm occurred after the second thrombectomy (white arrow); (C) frontal angiogram showing recanalization of the basilar artery after three-pass of stent retriever. Three months follow up: (D) the right vertebral artery did not converge with the basilar artery; (E) (F) re-established blood flow in the left vertebral and basilar arteries.

The cause of this patient is arterial to arterial embolism caused by arterial dissection. Considering these findings, endovascular treatment was as follows: a 6-French guiding catheter was introduced through a femoral sheath into the V2 segment of right VA. Then, a Rebar-18 microcatheter (ev3, Plymouth, MN) was deployed from the left P1 segment. Thrombectomy was performed through the thinner right VA with three passes of the Solitaire FR device 4 × 20 mm (Medtronic, Minneapolis, MN) in the BA. Vasospasm occurred after the second thrombectomy, which was significantly relieved after intra-arterial injection of nimodipine and tirofiban (Fig. 3). Subsequent follow-up angiograms showed successfully recanalized and reached modified treatment in cerebral ischemia score 3 of BA and left VA V4 segment still occluded. After thrombectomy, the consciousness of patients improved, and the National Institute of Health Stroke Scale score decreased to 12. The duration of the procedure was 1.5 hours.

Multiple infarcts of bilateral cerebellum, pons, and the left thalamus were found in the 24-hour magnetic resonance imaging (Fig. 1). Magnetic resonance angiography showed that the blood flow of the BA was normal, but the V4 segment of the left VA was occluded. Since no intracranial hemorrhage was found, low-molecular-weight heparin calcium 5000 U was subcutaneously injected twice a day for 25 days; therapy with warfarin to a target INR of range 2 to 3 continued for 2 months. Three months later, the patient has a modified Rankin Scale of 2, DSA showed re-established blood flow in the left vertebral and basilar arteries, and the dissection of left VA disappeared. There was no blood flow in the distal posterior inferior cerebellar artery of the right VA.

## 3 Discussion

EVT for selected patients with AIS caused by large vessel occlusions of the anterior circulation within 24 hours of onset has been proven safe and effective in multiple randomized controlled studies.^[2,4]^ As the posterior circulation was not supported from randomized trials, the current guidelines state that thrombectomy may be reasonable for carefully selected patients with AIS in whom treatment can be initiated (groin puncture) within 6 hours of symptom onset and who have causative occlusion of vertebrobasilar artery.^[5]^ The EVT for Acute Basilar Artery Occlusion Study (BASILAR) study shows that EVT can improve the rate of functional outcomes at 90 days by 3 times and a lower rate of 90-day mortality compared with the standard medical treatment alone.^[6]^ Similar to some previous studies using the new generation of stent-retrievers and aspiration device,^[7–10]^ our previous study^[11]^ which included 67 patients with vertebrobasilar artery occlusion found that the recanalization rate was 89.2%, 59.7% patients had favorable outcomes (modified Rankin Scale, 0−3), and the incidence of symptomatic intracranial hemorrhage was 4.5%. Since AIS caused by vertebrobasilar artery occlusion is a devastating subtype of stroke with high disability and mortality rates,^[12]^ embolectomy for this patient was performed.

From the DSA results of this patient, there was long-segment dissection of left VA V1 to V2 and the BA occlusion. Bonati et al^[13]^ suggested that artery-to-artery embolism is the main mechanism of stroke in cervical artery dissection, whereas others suggest that the main reason is hemodynamic failure at the distal of the dissection.^[14]^ The etiology of this case was considered to be the simultaneous presence of artery-to-artery embolism and hemodynamic origin, which was confirmed by the MRI 24 hours after the operation. Because of the different anatomic characteristics of posterior and anterior circulation, the choice of thrombectomy strategy was also different. The choice of thrombectomy strategy depends on the anatomic structure, accessibility, and the type and extent of the occlusive process.

In this case, although the left VA (dirty-road path) is the dominant supplying artery, it is difficult to find the true lumen and angioplasty due to the long-segment dissection of V1 to V2. This will delay the revascularization of the BA and the best time for treatment of the patient. Therefore, we chose the right VA (clean-road path) as the access site to perform a BA thrombectomy, without treatment of the extracranial left VAD. The problems that should be paid attention to in the process of EVT are as follows: vasospasm during thrombectomy due to the small diameter of blood vessels at the junction of right VA and BA; and rethrombosis of BA caused by untreated left vertebral dissection. The diameter of blood vessels at the junction of VA and BA is about 1.7 mm, less than the recommended vessel diameter available for Solitaire FR 4 × 20 mm (2 mm). The special crimped structure design of Solitaire has been reported to be used in thrombectomy with less than the diameter of 2 mm.^[15,16]^ The duration of acute anticoagulation is unclear; however, it may be inferred based on healing of the injured artery on follow-up imaging as deemed by the clinician on a case-by-case basis.^[17]^

Cerebral angiography performed three months post-operation showed that the right VA did not converge with the BA, but continued as the posterior inferior cerebellar artery. We speculate that this may be due to the left VA gradually recovering the dominant blood supply, and hemodynamically, the right VA on the nondominant side did not flow into the BA.

Anticoagulation therapy is an effective treatment for patients with extracranial VAD. When complicated with BA occlusion, choosing the clean-road path as the access site to perform a BA thrombectomy without treatment of the extracranial left VAD may be a fast and effective treatment strategy.

## Acknowledgments

We greatly appreciate the participating relevant cliniciansm, the imaging and laboratory technicians.

## Author contributions

**Conceptualization:** Changchun Jiang, Fei Hao, Yu Fan.

**Data curation:** Jiahui Liu, Yu Fan.

**Investigation:** Jinfeng Zhang, Yu Fan.

**Methodology:** Jinfeng Zhang, Cui Yujuan, Yu Fan.

**Resources:** Cui Yujuan, Jianqi Wei.

**Supervision:** Junfeng Yang.

**Visualization:** Junfeng Yang.

**Writing – original draft:** Jiahui Liu.

**Writing – review & editing:** Changchun Jiang, Yu Fan.
